# Effect of Nutrient Supplementation on the Biochemical Composition and Microbial Safety of Open-Pond Spirulina Cultivated in Cameroon

**DOI:** 10.3390/foods14173009

**Published:** 2025-08-28

**Authors:** Tchouli Noufeu, Tinghong Ming, Xiaoqun Zeng, Jiajie Xu, Mbezele Junior Yannick Ngaba, François Tchoumbougnang, Achille Njomoue Pandong, Salle Mpondo, Tchoulabi Keyeya, Moussa Gouife

**Affiliations:** 1 Microbial Development and Metabolic Engineering Laboratory, School of Marine Science, Ningbo University, Ningbo 315211, China; 2 State Key Laboratory for Quality and Safety of Agro-Products, College of Food Science and Engineering, Ningbo University, Ningbo 315211, China; 3 Department of Processing and Quality Control of Aquatic Products, Institute of Fisheries and Aquatic Sciences at Yabassi, University of Douala, Douala P.O. Box 7236, Cameroon; 4 Center of Molecular Ecophysiology (CMEP), College of Resources and Environment, Southwest University, Chongqing 400715, China; 5 National Advanced School of Maritime and Ocean Science and Technology (ENSTMO), University of Ebolowa, Kribi P.O. Box 292, Cameroon

**Keywords:** *Arthrospira platensis*, nutrient formulation, open-pond systems, microbial contamination, proximate composition, food hygiene, tropical aquaculture

## Abstract

*Spirulina* (*Arthrospira platensis*) is globally recognized for its high nutritional value and potential as a sustainable food source. However, the influence of targeted nutrient supplementation on its biochemical composition and microbial safety under tropical open-pond conditions remains underexplored, particularly in sub-Saharan Africa. This study evaluated the effects of three nutrient supplementation regimes (compositions A, B, and C) and a control on *Spirulina* cultivated over 30 days in raceway ponds at the Nomayos Spirulina Production Farm in Cameroon. All treatments maintained physicochemical parameters within ranges favorable for *Spirulina* growth. Composition A significantly enhanced protein content (60.38 ± 0.68%), while composition C promoted carbohydrate accumulation (28.02 ± 0.41%). Microbial assessments revealed variable contamination levels, with composition B exhibiting the highest *Escherichia coli* (1.05 ± 0.075 × 10^5^ CFU/g) and *Salmonella/Shigella* (4.09 ± 1.81 × 10^5^ CFU/g) counts, potentially due to nutrient-induced changes or post-harvest handling factors. Correlation analyses revealed a moderate positive relationship between nitrogen input and protein synthesis (r = 0.309), which was not statistically significant (*p* = 0.329). Additionally, higher pH was significantly correlated with total mesophilic counts (r = 0.661, *p* = 0.019) and *E. coli* (r = 0.655, *p* = 0.020). These findings highlight the importance of nutrient formulation and environmental management in improving nutritional quality while minimizing microbial risks during *Spirulina* cultivation in tropical, low-tech settings.

## 1. Introduction

Spirulina (*Arthrospira platensis*) is a filamentous cyanobacterium recognized for its rich nutritional profile, which encompasses high-quality proteins, essential amino acids, vitamins, minerals, and various bioactive compounds. It contains approximately 60–70% protein by dry weight, alongside essential fatty acids and micronutrients such as iron, calcium, and zinc, highlighting its potential benefits as a dietary supplement and functional food [1,2,3]. Due to its exceptional nutrient density and associated health-promoting properties, Spirulina has gained global attention as a functional food, particularly in developing regions that strive to combat food insecurity and malnutrition [4,5,6]. While comprehensive studies on Spirulina farming in Cameroon remain limited, its increasing interest among small-scale producers suggests potential for sustainable and locally adapted production systems [7].

The cultivation of Spirulina typically occurs in open-pond systems, which are subject to natural environmental conditions that significantly affect growth performance and biomass safety. Critical factors influencing Spirulina production include nutrient availability and environmental parameters such as temperature, pH, salinity, and turbidity, which are vital in determining biomass yield and biochemical composition while also posing risks for microbial contamination particularly in environments with suboptimal water quality and exposure to external contaminants [8,9]. Nutrient modulation, particularly the balance of minerals and nitrogen sources, can significantly impact the metabolic pathways of Spirulina, resulting in variations in its protein, carbohydrate, lipid, and ash content [10,11]. Consequently, optimizing nutrient supplementation is essential for maximizing the nutritional quality of Spirulina biomass [12].

Despite the low-cost advantage of open-pond cultivation for Spirulina, exposure to microbial contamination from environmental sources poses significant food safety challenges, particularly in artisanal systems lacking sanitation controls [13,14]. Contaminants may include pathogenic bacteria such as *Escherichia coli* (*E. coli*) and *Salmonella* spp., particularly alarming in regions with limited access to advanced processing and hygiene controls [15]. Additionally, cyanotoxins such as microcystins, produced by contaminating cyanobacterial strains, represent a critical food safety concern in open-pond Spirulina cultivation systems [16]. Therefore, ensuring the microbiological safety of Spirulina biomass is paramount to consumer protection and compliance with regulatory standards [17,18]. Contaminated Spirulina products may pose health risks, particularly if not properly processed or dried, necessitating strict monitoring of cultivation practices and product safety [19].

Previous studies from Chad and Kenya have documented both traditional and small-scale industrial production of Spirulina in Sub-Saharan Africa. In Chad, Spirulina has been harvested from Lake Kossorom and processed into dry biomass for household consumption and income generation [20]. In Kenya, community-based Spirulina cultivation projects have supplied the product for health and nutrition programs supported by public and private health organizations [21]. However, these studies have primarily focused on cultivation practices and social benefits, with limited emphasis on the biochemical composition or microbial safety of Spirulina produced under semi-controlled, open-pond conditions.

Building on these regional insights, there is a pressing need for improved monitoring and standardization of Spirulina production worldwide. Variability in cultivation methods, environmental conditions, and post-harvest handling leads to inconsistent product quality and complicates the establishment of universal safety guidelines [15]. While nutrient availability, particularly nitrogen and minerals, plays a key role in shaping Spirulina’s biochemical profile, emerging evidence suggests that excessive or imbalanced nutrient supplementation may promote microbial growth, particularly under conditions of high turbidity or poor aeration [22,23]. Moreover, the extent to which nutrient modulation might mitigate or exacerbate microbial risks, including the proliferation of pathogens, remains unclear and warrants further investigation [24,25].

To address these knowledge gaps, the present study conducts a thorough comparative analysis of Spirulina biomass produced under three distinct mineral supplementation regimens. This research aims to evaluate the impact of various nutrient supplementation regimens on the nutritional quality and microbiological safety of Spirulina cultivated under semi-controlled, open-pond conditions in Cameroon. Specifically, the study seeks to: (1) assess the effects of nutrient supplementation on the biochemical composition of Spirulina, (2) evaluate microbiological contamination risks associated with each nutrient treatment, and (3) analyze correlations between nutrient inputs and environmental factors with Spirulina biochemical and microbial profiles. To our knowledge, this is among the first Sub-Saharan African studies to jointly examine nutritional quality and microbial safety of Spirulina under semi-artisanal conditions, providing new insights for safe, sustainable production in the region.

## 2. Materials and Methods

### 2.1. Study Site and Cultivation Conditions

The study was conducted at the Nomayos Spirulina Production Farm in Cameroon, located approximately 20 km from Yaoundé at coordinates 3°46′60″ N, 11°22′60″ E. This site is a leading artisanal spirulina production facility operating under semi-controlled, open-air conditions. Cultivation was carried out in nine identical raceway ponds constructed from reinforced concrete and lined with food-grade polyethylene tarpaulin. Each pond measured 4.0 m in length, 1.25 m in width, and 0.3 m in depth, maintaining an effective culture volume of 1000 L at a consistent level of approximately 20 cm to optimize light penetration and mixing. The water used for medium preparation was sourced from a constructed well located on-site. Prior to use, the water underwent a filtration process designed to ensure its suitability for Spirulina cultivation. The initial pH of the medium was adjusted to 9.0 using sodium carbonate to create optimal conditions for Spirulina growth, as this pH level has been shown to support maximum biomass productivity and minimize contamination risks. Circulation was provided by manually operated paddle wheels rotating at 12–15 rpm, sufficient to prevent sedimentation and maintain homogeneous suspension.

Three ponds were assigned to each of the three nutrient supplementation regimes (compositions A, B, and C), while an additional three ponds were designated as the control group. The control group did not receive any daily nutrient supplementation but was otherwise treated identically to the experimental groups. This included daily water addition, stirring to ensure uniformity, and exposure to natural light and ambient temperature, providing a baseline for comparison. All ponds were seeded with the same standardized mineral inoculation medium (Table 1) to ensure identical starting conditions.

The cultivation period lasted 30 days, with samples collected at three key time points (Days 10, 20, and 30). These time points were selected to represent critical phases of the Spirulina growth cycle: early growth (Day 10), mid-growth (Day 20), and peak biomass (Day 30). This sampling strategy allowed us to capture significant changes in biomass quality and microbial safety throughout the cultivation period. Samples were collected independently from each of the three ponds per treatment group and analyzed separately, yielding three biological replicates per condition and time point.

### 2.2. Sample Collection and Processing

Samples were collected from the three ponds assigned to each treatment group (compositions A, B, C, and Control) at three distinct time points during the cultivation period: Day 10 (early growth), Day 20 (mid-growth), and Day 30 (peak biomass). At each time point, five subsamples were collected from each pond, taken from equidistant locations (center and four corners) to ensure representativeness. The fresh biomass was filtered, collected, and processed hygienically. Spirulina was pressed using a sterilized cloth system between two grooved wooden boards. The paste was extruded into thin strands and dried on gas-heated trays at 45–50 °C for 3 h. After drying, the material was finely ground using a sterile mixer. The resulting powder was vacuum-packed and stored at −20 °C until further analysis. All microbiological analyses were conducted within 6 h of collection to preserve microbial integrity, while biochemical analyses were performed within one week of storage to ensure nutrient stability. Bromatological assays were performed using the Spirulina biomass from each group.

### 2.3. Monitoring Physicochemical Parameters in Spirulina Cultivation

To characterize the physicochemical environment during spirulina cultivation, five key parameters, temperature, pH, density, turbidity (Secchi depth), and salinity, were monitored daily in each 1000 L culture basin over 30 days. Measurements were conducted each morning between 6:30 a.m. and 8:30 a.m. from a consistent central location to ensure representative and reproducible sampling. The water temperature was measured using a calibrated digital thermometer that was immersed directly in the culture medium. The pH was measured daily using universal indicator strips (Merck, Darmstadt, Germany) and cross-verified weekly using a calibrated benchtop pH meter. Density was measured with a precision glass hydrometer using samples collected in graduated cylinders. Turbidity was assessed using the Secchi disk, which was immersed until it disappeared from view. Salinity (g/L) was estimated indirectly using the observed density (DT) and temperature (T), following the equations:

(1) D_20_ = (T − 20) × 0.325 + DT,

(2) Salinity = (D_20_ − 1007.6) × 1.250 +10,

where *D*_20_ is the density corrected to 20 °C, and the constants were derived from empirical calibrations for tropical freshwater systems.

### 2.4. Microscopic Morphological Evaluation

To evaluate the structural integrity, physiological status, and purity of Spirulina cultures, microscopic observations were conducted on samples from each cultivation group on the 30th day. A drop of fresh culture was placed on a clean glass slide, covered with a coverslip, and examined under a standard optical light microscope. Morphological traits of Spirulina filaments, including shape (spiral, straight, or aggregated), length (short if <100 µm or long if >100 µm), and the number of spirals per filament (2–4 or >5), were assessed at 40× magnification. Vitality was determined based on filament motility, structural integrity, and pigment intensity (green coloration). Additionally, microbial purity was assessed at 400× magnification by examining five randomly selected fields of view per slide. The presence of extraneous microorganisms, including bacteria, protozoa, non-*Arthrospira* cyanobacteria, and contaminant algae, was recorded using a semi-quantitative scoring system: (−) for absence, (±) for low or sporadic presence (1–2 occurrences per field), and (+) for high or widespread presence (≥3 occurrences per field). This scoring method provided a consistent basis for evaluating microbial contamination across treatment groups.

### 2.5. Bromatological Quality Assessment

The proximate composition of dried Spirulina samples was determined using standard methods to quantify moisture, dry matter, crude protein, total lipids, ash, and carbohydrates. All analyses were conducted in triplicate, with each value derived from independently sampled and pooled pond cultures within the same nutrient group.

Moisture content was determined gravimetrically following AFNOR [26] procedures. Approximately 5 g of Spirulina sample (m_1_) was placed in pre-weighed crucibles (m) and dried in a convection oven at 105 °C for 1.5 h until constant weight was achieved. After cooling in a desiccator, the final mass (m_2_) was recorded. Moisture content was calculated as:

(3) Moisture content (%) = [(m1 − m2)/(m1 − m)] × 100,

Dry matter content was obtained by difference:

(4) Dry matter (%) = 100 − Moisture content,

Lipid extraction was performed using a Soxhlet apparatus in accordance with IUPAC guidelines [27]. Fifteen grams of Spirulina biomass (P_1_) were placed into cellulose extraction thimbles. Approximately 300 mL of hexane was used as the solvent under reflux at 65 °C for 8 h. The lipid extract was concentrated using a rotary evaporator, and the residue was weighed (P_3_). The flask weight prior to extraction was recorded as P_2_. Lipid content was calculated as:

(5) Lipid content (%) = [(P3 − P2)/P1] × 100,

Crude protein content was quantified using the Kjeldahl method per AOAC guidelines [28]. To begin the process, approximately 0.10 g of Spirulina biomass was digested in a mixture of concentrated sulfuric acid, salicylic acid, and a blend of copper sulfate, potassium sulfate, and selenium until the solution became clear. Following digestion, the digest was alkalinized with 40% sodium hydroxide and subjected to steam distillation using an automated Kjeldahl unit (BUCHI 315). During this process, released ammonia was captured in 20 mL of boric acid solution containing mixed indicators (methyl red and bromocresol green) and titrated with 0.01 N hydrochloric acid (HCl) until the endpoint color shifted from green to violet. Additionally, a reagent blank was processed in parallel to correct for background nitrogen. Finally, nitrogen content was calculated using the following equation:

(6) Nitrogen (%) = [(V1 − V0) × N × 14.007]/Sample mass (g),

where *V*_1_ and *V*_0_ are the volumes of HCl used for the sample and blank, respectively, and *N* is the normality of HCl. The protein content was then determined using a nitrogen-to-protein conversion factor of 6.25:

(7) Protein (%) = Nitrogen (%) × 6.25,

Ash content was measured using the incineration method described by AOAC. Approximately 1.50 g of dried sample was placed in a pre-weighed porcelain crucible (P_1_) and incinerated in a muffle furnace at 560 °C for 4 h. After cooling in a desiccator, the final weight (*P*_3_) was recorded. *P*_2_ represents the weight of the crucible plus sample before incineration.

(8) Ash content (%) = [(P3 − P1)/(P2 − P1)] × 100,

Total carbohydrate content was estimated by difference, according to standard proximate analysis methodology:

(9) Carbohydrates (%) = 100 − (Moisture + Protein + Lipids + Ash),

This method assumes that the remaining fraction of the dry matter consists primarily of carbohydrates and non-nitrogenous organic compounds.

### 2.6. Microbiological Quality Assessment

Microbiological analysis was performed on dehydrated Spirulina powder to assess total microbial load and detect potential contamination by specific pathogens. For each sample, 10 g of Spirulina powder was aseptically homogenized in 90 mL of peptone buffered water using a magnetic stirrer for 2 min. The suspension was left at room temperature for 30 min to facilitate microbial revival. A series of decimal dilutions, ranging from 10^−1^ to 10^−7^, was prepared using sterile physiological saline, in accordance with conventional microbiological procedures to ensure accurate enumeration across a broad spectrum of microbial densities. Three selective and differential media were used to enumerate microbial populations and detect pathogens: Plate Count Agar (PCA) for total aerobic mesophilic counts, MacConkey Agar for Gram-negative enterobacteria, including Escherichia coli, and Salmonella–Shigella (SS) Agar for the presumptive identification of *Salmonella* spp. and *Shigella* spp. Media were prepared according to the manufacturer’s protocols (BD, Franklin Lakes, NJ, USA) and following the international standard method ISO 6579-1 [29]; they were autoclaved at 121 °C for 15 min, and then poured into sterile 90 mm Petri dishes under aseptic conditions (Table 2).

To assess the total number of aerobic mesophilic bacteria, 10 μL aliquots from the prepared dilutions were plated onto PCA using the spread plate method. Plates were incubated aerobically at 35 °C for 24 h. Colony-forming units were counted manually, and results were expressed as CFU/g using the formula:

(10) CFU/g = Nt × n,

where Nₜ is the number of colonies, and n is the dilution factor.

*E. coli* detection was carried out by directly plating the undiluted and diluted samples onto MacConkey Agar, followed by incubation at 37 °C for 24 h. Colonies that appeared red or pink with bile precipitate (indicative of lactose fermentation) were considered presumptive *E. coli*. For the detection of *Salmonella* and *Shigella*, initial screening was also performed on MacConkey Agar, with non-lactose-fermenting colonies (colorless or pale) being subcultured onto SS Agar for further identification. On SS Agar, black-centered colonies were considered presumptive *Salmonella* (due to H_2_S production), while pale colonies lacking H_2_S production were indicative of *Shigella* spp.

Guidelines from the International Commission on Microbiological Specifications for Foods and the World Health Organization (WHO) specify acceptable limits for pathogenic bacteria, such as a zero *Salmonella* count in 25 g of food and *E. coli* levels not exceeding 100 CFU/g in products intended for human consumption [30,31]. These thresholds are essential for assessing the microbiological safety of Spirulina biomass and ensuring public health protection.

### 2.7. Statistical Analysis and Software

All statistical analyses and visualizations were performed using R software (version 4.4.1; R Core Team, Vienna, Austria). Mean values and standard deviations were calculated for each set of experimental data using base R and the dplyr package. Prior to performing Pearson correlation analyses, normality of the variables was assessed using the Shapiro–Wilk test, and the independence of observations was assumed based on the experimental design, where samples were collected and processed independently. Pearson correlation coefficients were computed using the cor.test() function to investigate the impact of nutrient inputs and environmental conditions on the bromatological and microbiological quality of Spirulina, assuming linear relationships and normal distributions of the variables. Multivariate visualizations, including radar plots, z-score bar charts, and correlation heatmaps, were generated using the ggplot2, fmsb, and corrplot packages. Differences were considered statistically significant at *p* < 0.05. For comparative analysis, raw values were standardized using z-score normalization to allow proportional interpretation across parameters with different scales. The z-score was calculated as:

(11) Z = (X − μ)/σ,

where X is the observed value, μ is the mean, and σ is the standard deviation across the monitoring period.

## 3. Results

### 3.1. Physicochemical Conditions and Temporal Variability of Spirulina Cultivation

The water temperature remained stable at 23.45 ± 0.80 °C, within the optimal range for Spirulina growth (Table 3). The pH was measured as alkaline at 10 ± 0.10, promoting cyanobacterial proliferation while limiting contaminants. Liquid density averaged 1015.87 ± 2.39 g/L, with minor fluctuations. Water transparency, as estimated by Secchi disk depth, averaged 4.57 ± 1.11 cm, indicating a moderate increase in suspended biomass. Salinity varied between 17.0 and 24.1 g/L, with a mean of 20.16 ± 2.03 g/L, likely due to evaporative concentration.

**Figure 1:**
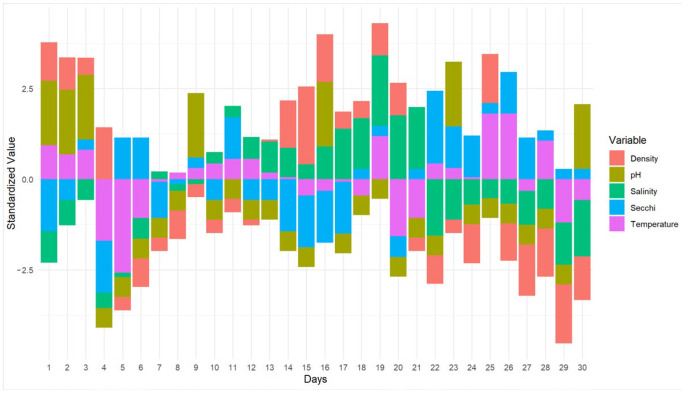
Z-score standardized values of physicochemical parameters.

A standardized z-score stacked bar chart was used to evaluate temporal variability (Figure 1), highlighting the stability of temperature and pH throughout the experimental period. In contrast, salinity and density exhibited more pronounced fluctuations, particularly in the latter half of the cycle, suggesting cumulative evaporative effects. The Secchi depth exhibited intermittent variation, possibly indicating fluctuations in water clarity and cell concentration resulting from growth cycles or sediment resuspension. Overall, the monitored conditions remained favorable for Spirulina cultivation, emphasizing the importance of controlling evaporation to maintain stable salinity and optimize production.

### 3.2. Morphological and Microbial Profiling of Spirulina Cultures

**Figure 2:**
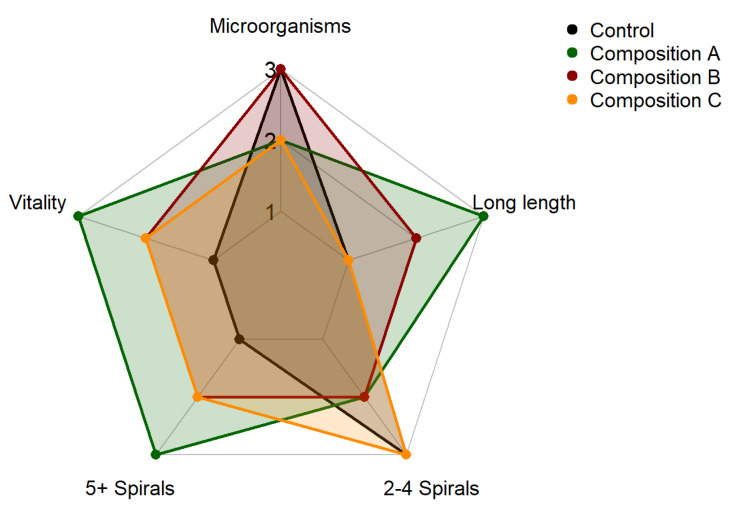
Radar plot illustrating the morphological integrity and microbial presence of Spirulina under three nutrient compositions (A, B, C) and control.

After 30 days of cultivation, microscopic assessment of Spirulina revealed distinct differences in structural integrity and microbial presence among the three nutrient compositions and the control (Figure 2). Spirulina cultivated under composition A exhibited the most favorable morphological characteristics, including high vitality, an abundance of long filaments, and a greater proportion of filaments with five or more spirals, reflecting robust physiological development and structural integrity. In contrast, the control group demonstrated the weakest morphology, characterized by low vitality, predominantly short filaments, and poor spiral development, indicating nutrient limitation and suboptimal cellular differentiation. Spirulina cultured with composition B showed relatively high vitality but only moderate morphological features. It also exhibited the highest microbial contamination levels, which may be associated with nutrient imbalances in this formulation, potentially fostering conditions favorable for bacterial proliferation. Composition C presented a more balanced profile, with moderate vitality, good spiral formation, primarily consisting of 2–4 spirals, and a lower microbial contamination score, although the occurrence of long filaments remained limited. These findings underscore the critical role of nutrient formulation in shaping the morphological quality and microbial safety of Spirulina biomass, highlighting how targeted supplementation enhances structural development while influencing contamination risk.

### 3.3. Proximate Composition of Spirulina Under Different Nutrient Regimes

The proximate composition of Spirulina biomass was monitored across three cultivation periods under different nutrient formulations and control. As shown in Table 4, all groups exhibited progressive biochemical changes over time, with distinct patterns according to nutrient composition. Composition A resulted in the highest protein accumulation, increasing from 51.80 ± 0.70% on Day 10 to 60.38 ± 0.68% on Day 30. Conversely, composition C showed a marked increase in carbohydrate content, from 22.90 ± 0.52% to 28.02 ± 0.41% over the same period, suggesting a possible nitrogen-limited metabolic shift. Lipid content declined in all groups with time but remained highest in composition A (3.07 ± 0.19% on Day 30), while composition C recorded the lowest lipid values throughout. Ash content remained relatively stable across groups and time points (18.90–20.11%). The control group exhibited intermediate trends, with moderate increases in protein (49.20 ± 0.55%) and carbohydrates (24.10 ± 0.47%) by Day 30. These findings highlight the significant impact of nutrient modulation on the biochemical composition of Spirulina, demonstrating that composition A enhances protein content, while composition C promotes carbohydrate accumulation. This suggests that tailored nutrient regimes can be strategically employed to enrich Spirulina biomass with specific macronutrients according to targeted nutritional or industrial objectives.

### 3.4. Microbiological Contamination Profile of Spirulina Biomass Across Nutrient Formulations

Microbiological profiling of Spirulina biomass cultivated under open-air, semi-controlled conditions is presented in Table 5 and reveals a progressive accumulation of microbes over time, with the total mesophilic counts (TMC) increasing across all treatments. By Day 30, TMC ranged from 4.5 ± 1.47 × 10^6^ CFU/g in composition B to 6.1 ± 4.29 × 10^6^ CFU/g in composition C, while the control and composition A registered intermediate levels (4.7 ± 0.89 and 5.0 ± 0.32 × 10^6^ CFU/g, respectively). Although composition B displayed relatively lower TMC, it was disproportionately associated with elevated levels of enteric pathogens. *E. coli* contamination was detected in all groups, escalating over time and peaking in composition B at 1.05 ± 0.075 × 10^4^ CFU/g by Day 30, underscoring potential hygiene concerns. Notably, composition B also exhibited the highest levels of *Salmonella* and *Shigella*, with counts reaching 4.09 ± 1.81 × 10^2^ CFU/g and 5.92 ± 3.15 × 10^2^ CFU/g, respectively, raising critical concerns for microbial safety. In contrast, composition A maintained the lowest *Shigella* load (0.30 ± 0.40 × 10^2^ CFU/g) and moderate *Salmonella* contamination (2.07 ± 0.71 × 10^2^ CFU/g), indicating a comparatively more stable microbiological profile under nutrient-driven modulation. These findings underscore the influence of specific nutrient regimes not only on the total microbial load but also on the varying levels of pathogenic contamination observed in Spirulina biomass cultivated under artisanal conditions.

### 3.5. Correlation of Nutrient Inputs and Environmental Factors with Spirulina Biochemical and Microbial Profiles

**Figure 3:**
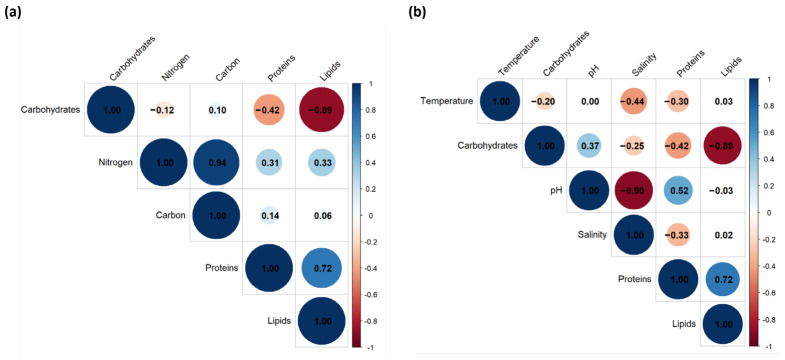
Pearson correlation analysis of nutrient inputs (**a**) and environmental factors (**b**) with biochemical profiles of Spirulina.

To elucidate the drivers of biochemical variability in Spirulina, Pearson correlation analyses were conducted between nutrient inputs (total nitrogen and carbon), environmental parameters (temperature, pH, salinity), and key bromatological components (proteins, carbohydrates, lipids). As illustrated in Figure 3, nitrogen showed a weak positive correlation with protein content (r = 0.309, *p* = 0.329), and lipid content (r = 0.335, *p* = 0.288). Although these trends suggest potential nutrient-mediated enhancement of biosynthetic pathways, the associations were not statistically significant and should be interpreted cautiously. The lack of significant correlation between carbon and carbohydrate or protein content highlights a limitation in our understanding, as we cannot conclusively determine the role of carbon availability from these data alone. Among environmental variables, pH showed a moderate positive correlation with protein content (r = 0.518, *p* = 0.084), suggesting that more alkaline conditions might favor enzymatic activity involved in nitrogen assimilation and protein synthesis, although this relationship also lacked statistical significance. Salinity was negatively correlated with protein content (r = –0.335, *p* = 0.288), hinting at a possible stress-related reduction in biomass quality under saline conditions. None of the environmental parameters showed significant correlations with lipid or carbohydrate levels, suggesting relative biochemical resilience to environmental fluctuations within the monitored ranges.

**Figure 4:**
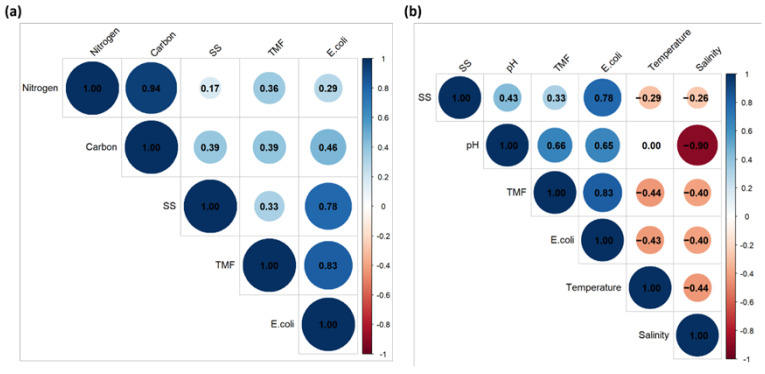
Pearson correlation analysis of microbial indicators with nutrient inputs (**a**) and environmental factors in Spirulina (**b**).

Figure 4 illustrates how key cultivation variables influenced microbial safety in Spirulina biomass through Pearson correlation analyses between microbial indicators (TMC, *E. coli*, and *Salmonella*/*Shigella*) and nutrient/environmental parameters. TMC showed moderate positive, but non-significant, correlations with both nitrogen and carbon inputs. Similarly, *E. coli* levels were moderately correlated with carbon, though weakly with nitrogen, without reaching statistical significance. Notably, pH was significantly positively correlated with TMC (r = 0.661, *p* = 0.019) and *E. coli* (r = 0.655, *p* = 0.020), indicating that more alkaline environments may favor microbial proliferation. Salinity displayed non-significant negative correlations with all microbial indicators, suggesting possible microbial suppression under higher ionic conditions. Temperature was also negatively correlated with TMC and *E. coli*, though the associations were not statistically significant.

## 4. Discussion

### 4.1. Nutrient Modulation and Its Impact on Spirulina’s Biochemical Composition

The nutritional quality of Spirulina is strongly influenced by the availability of nutrients, particularly nitrogen and carbon sources. In this study, although Pearson correlation analysis indicated a weak and statistically non-significant relationship between nitrogen input and protein content across all treatments (r = 0.309, *p* = 0.329), Composition A, which featured the highest nitrogen input, still produced the highest protein content (60.38 ± 0.68%) by Day 30. This discrepancy can be attributed to the fact that correlation analysis captures overall trends across all data points and time frames, potentially diluting specific treatment effects. The observed increase in protein content in Composition A suggests a more complex, possibly nonlinear relationship influenced by the highest nitrogen input in this specific formulation, which may not be fully captured by linear correlation analysis. Numerous studies have demonstrated that nitrogen availability is a primary driver of both biomass productivity and protein accumulation in Spirulina cultures. For instance, Spirulina maxima achieved protein levels between 60.1% and 71.8% when grown in nutrient-rich media supplemented with fermented cattle and poultry manure, highlighting the role of nutrient enrichment in boosting protein yield [32]. Similarly, optimization of nitrogen concentration and cultivation time has been shown to maximize biomass and protein extraction [33]. The type of nitrogen source can also influence outcomes, with urea outperforming potassium nitrate in enhancing protein content due to its higher nitrogen uptake efficiency [34,35]. Reported protein contents under favorable conditions range from approximately 58.2% to 69.2% [3], further underscoring nitrogen’s pivotal role in protein biosynthesis.

In addition to nitrogen-driven protein synthesis, carbon availability, particularly as bicarbonate, significantly influences Spirulina’s carbohydrate accumulation. Composition C, characterized by the highest bicarbonate concentration, yielded the highest carbohydrate content (28.02 ± 0.41%). This finding substantially exceeds the 6.46 ± 0.32% reported by Seghiri et al. in a Moroccan Spirulina strain, which itself was noted as below the common 15–25% range for many species [36]. This variation underscores the combined effects of strain specificity and environmental factors on biochemical profiles. Our findings align closely with Andrade et al., who documented a similar carbohydrate concentration (29.03%) in Spirulina, reinforcing its potential as a superior carbohydrate source compared to other microalgae like *Chlamydomonas reinhardtii* (4.99%) [37]. Moreover, Koli et al. reported carbohydrate and protein contents of 21.87% and 65.71%, respectively, demonstrating the typical balance of macronutrients in Spirulina that supports its use as a nutritionally versatile supplement, especially for applications requiring simultaneous protein and energy enhancement [38].

Lipid dynamics within Spirulina were also notably affected by nutrient inputs. Spirulina cultivated under composition A maintained the highest lipid levels (3.07 ± 0.19%) throughout the cultivation cycle, contrasting with the observed decline in lipid content across all treatments over time. The sustained lipid retention in this scenario can be attributed to the synergistic impact of abundant nitrogen and adequate phosphorus, which support membrane lipid biosynthesis while mitigating oxidative degradation, particularly in open-air cultivation environments [39,40]. In contrast, despite the carbohydrate accumulation observed in composition C, it consistently recorded the lowest lipid content (1.36 ± 0.23% on Day 30), suggesting a metabolic trade-off where excess carbon is preferentially stored as polysaccharides rather than lipids. This phenomenon is frequently noted in cyanobacteria under nitrogen stress conditions, where carbon is diverted from lipid synthesis to other metabolic pathways [41].

These results underscore the potential for optimizing nutrient regimes to tailor Spirulina’s nutritional profile to meet specific industrial objectives. The production of high-protein Spirulina, as achieved with composition A, is invaluable for functional foods and dietary applications. While the carbohydrate-rich biomass from composition C may hold promise for applications such as bioethanol production or as a component in energy bars, further analysis of digestibility and fermentable sugar content is required to confirm its suitability for these uses [38,42]. Moreover, the relative stability of ash content across the treatments (ranging from 18.90 to 20.11%) indicates that mineral accumulation remained largely unaltered, further suggesting the primary influence of nitrogen-carbon allocation over osmotic mineral stress [43]. This metabolic plasticity reinforces the versatile potential of Spirulina as a customizable platform for sustainable and nutrient-rich biomass production.

### 4.2. Microbiological Risk Under Open-Pond Conditions

The microbiological profiling of Spirulina cultivated under different nutrient regimes provides critical insights into the contamination dynamics inherent to open-pond systems. Across all experimental groups, TMC exhibited a progressive increase over time, a trend consistent with previous studies that have demonstrated the risk of microbial proliferation due to biofilm formation and exposure to environmental contaminants [44,45]. By Day 30, TMC levels ranged from 4.5 × 10^6^ to 6.1 × 10^6^ CFU/g, values comparable to those observed in other artisanal Spirulina systems [46,47,48]. Notably, composition C reached the highest TMC level (6.1 ± 4.29 × 10^6^ CFU/g), indicating substantial microbial colonization. The significant variability observed in composition C (SD = 4.29) may reflect heterogeneity in pond exposure, underscoring the challenges of maintaining microbiological safety in open-pond cultivation systems.

*E. coli* contamination was detected in all groups, with levels escalating over time and peaking in composition B (1.05 ± 0.075 × 10^4^ CFU/g by Day 30). These findings are concerning, as they exceed the acceptable thresholds for dried algal foods set by various food safety authorities [49]. The prevalence of *E. coli* in these samples suggests potential hygiene issues and highlights the need for improved cultivation practices to minimize the risk of fecal contamination. Composition B was particularly affected, not only registering the highest *E. coli* levels but also exhibiting the most alarming values of *Salmonella* (4.09 ± 1.81 × 10^2^ CFU/g) and *Shigella* (5.92 ± 3.15 × 10^2^ CFU/g). These values far surpass internationally accepted food microbiological criteria, underscoring the heightened risk of pathogen amplification under nutrient regimes that may favor enteric microbial proliferation, potentially due to an insufficient antimicrobial mineral balance [50].

Interestingly, composition A demonstrated a more favorable microbial profile, with consistently lower pathogen counts than other treatments by Day 30. Despite an increase in TMC, *Salmonella* and *Shigella* levels remained below critical thresholds, suggesting that the specific nutrient formulation of composition A may contribute to conditions that limit pathogenic colonization. This aligns with previous reports highlighting the potential of specific nutrient conditions, such as adequate phosphate and bicarbonate concentrations, to create unfavorable environments for enteric bacteria [51]. Meanwhile, the control group, although untreated, displayed intermediate contamination levels, underscoring the contribution of environmental factors such as dust, insects, birds, and human activity to baseline contamination in artisanal systems [15,16].

In Cameroon, specific national microbiological standards for Spirulina are not formally established. However, local producers and health agencies generally refer to international benchmarks such as the Codex Alimentarius guidelines and the WHO standards. These standards typically require the absence of *Salmonella* in 25 g, *E. coli* levels below 10^2^ CFU/g, and TMC below 10^6^ CFU/g for dried algal products to ensure product safety for human consumption. Based on these criteria, the Spirulina biomass harvested under Compositions B and C exceeded acceptable levels, particularly in *E. coli*, *Salmonella*, and *Shigella*. Only Composition A approached compliance by maintaining low pathogen levels by Day 30, although TMC slightly exceeded the general benchmark. These findings highlight the necessity for enhanced hygiene and sanitation practices in Spirulina cultivation to ensure compliance with local and international food safety regulations.

### 4.3. Environmental Parameters as Modulators of Spirulina’s Functional Traits

The physicochemical environment during cultivation plays a pivotal role in shaping the biochemical and microbial attributes of Spirulina biomass. Among the monitored parameters, pH emerged as a key modulator, demonstrating a moderately strong positive correlation with protein content (r = 0.518, *p* = 0.084). This observation aligns with the known influence of alkaline pH in enhancing the solubility of inorganic nitrogen species and activating nitrogen assimilation pathways, particularly those involving Rubisco carboxylase and glutamine synthetase [52,53]. Alkaline conditions have been reported to favor the incorporation of ammonium and nitrate into amino acid biosynthesis, ultimately driving protein accumulation in cyanobacteria [54,55]. The relatively stable pH (10.0 ± 0.10) observed throughout the study likely contributed to the high protein yields, especially in nutrient-rich compositions such as A. However, excessive alkalinity may also introduce risks, particularly in open systems where natural buffering capacity is limited and microbial dynamics are more complex.

Salinity, another critical environmental parameter, exhibited a negative correlation with protein content (r = −0.335, *p* = 0.288), indicating a potential stress response that redirects metabolic energy away from protein biosynthesis. This finding is consistent with earlier studies highlighting the inhibitory effects of salt stress on enzymatic activity, nitrogen uptake, and amino acid synthesis in Spirulina cultures [56,57]. The progressive increase in salinity observed during the later stages of cultivation, likely due to cumulative evaporation, may have contributed to reduced protein yields in compositions with lower nitrogen buffering. Furthermore, the salinity-induced osmotic stress could partially explain the higher accumulation of carbohydrates observed in composition C, as cyanobacteria are known to accumulate compatible solutes and polysaccharides as a protective response [58].

Notably, environmental parameters also impacted microbial safety outcomes. pH showed a significant positive correlation with microbial indicators, particularly TMC (r = 0.661, *p* = 0.019) and *E. coli* (r = 0.655, *p* = 0.020), suggesting that high pH conditions facilitate the growth of Spirulina while concurrently promoting the proliferation of opportunistic pathogens like *E. coli* in nutrient-rich aquaculture environments. The dual effect of high pH is underscored by findings from studies that show microorganisms, especially Gram-negative bacteria, possess mechanisms that allow them to tolerate and exploit alkaline conditions [59,60]. Effective pH regulation is crucial for maintaining ecological balance in open-pond systems, as fluctuations can shift microbial community structures, favoring pathogenic over beneficial organisms and potentially compromising safety [61,62].

### 4.4. Implications for Artisanal Spirulina Production and Public Health

The significance of artisanal Spirulina production in Cameroon, conducted under semi-controlled, open-air conditions, is substantial. This approach promotes sustainable food systems using simple, cost-effective methods that align with local agricultural practices. Open-pond systems, which are easy to maintain and require minimal infrastructure compared to controlled indoor systems, present a viable option for small-scale farmers and artisanal producers [63]. A crucial aspect of this production method involves optimizing nutrient regimes to enhance the protein-rich biomass yield of Spirulina. Its reputation as a high-protein feed is well established, and tailored nutrient supplementation can significantly boost productivity. While regions like Cameroon face challenges related to malnutrition and protein deficiency [20,21], it is important to note that this study does not directly evaluate the impact of Spirulina production on food security or consumer nutritional outcomes. However, by adapting nutrient inputs to local conditions, producers might better meet local nutritional needs, although further research is needed to assess the direct benefits on food security and public health.

However, the positive aspects of this artisanal approach must be weighed against the study’s finding of elevated pathogen levels under specific nutrient regimes, which presents a critical challenge to food safety. Ensuring the microbial safety of Spirulina is essential for human consumption [64]. To address this issue, implementing strict hygiene protocols during production and post-harvest handling is necessary to minimize pathogen risks. These practices should include regular cleaning, proper biomass handling and storage, and possibly the use of antimicrobial treatments to safeguard the end product [47]. Furthermore, continuous monitoring of physicochemical parameters, such as salinity and temperature, is crucial for optimal Spirulina growth, as variations in these parameters can significantly impact biomass yield and quality [65]. Given Cameroon’s climate variability, producers need to adopt a proactive monitoring strategy to make timely adjustments that mitigate microbial risks and ensure consistent, high-quality Spirulina production.

Nevertheless, this study has limitations that should be acknowledged. First, microbial assessments were limited to indicator pathogens and did not include a broader microbial community analysis, which could provide deeper insights into contamination sources or microbial dynamics. Second, while temperature and salinity were monitored, environmental fluctuations inherent to open-air systems were not fully controlled, which could introduce variability in outcomes. Finally, although good handling practices were recommended, post-harvest processing steps were not experimentally evaluated, which restricts the conclusions we can draw regarding contamination risks during the downstream phase.

## 5. Conclusions

This study demonstrates that nutrient supplementation has a decisive influence on the biochemical and microbiological profiles of Spirulina cultivated under semi-controlled open-pond conditions in Cameroon. Composition A, with the highest nitrogen inputs, significantly boosted protein content (60.38 ± 0.68%) and lipid content (3.07 ± 0.19%) by Day 30. In contrast, composition C, rich in carbon but limited in nitrogen, favored carbohydrate accumulation (28.02 ± 0.41%) and improved morphological uniformity. Although all treatments supported satisfactory biomass growth, microbial assessments revealed important safety concerns. Notably, composition B was associated with elevated contamination levels, particularly *Salmonella* (4.09 ± 1.81 × 10^2^ CFU/g) and *Shigella* (5.92 ± 3.15 × 10^2^ CFU/g), by Day 30. In several instances, microbial counts, especially for *E. coli*, *Salmonella* and TMC, exceeded international food safety thresholds for dried products as defined by the WHO and Codex Alimentarius standards. These findings raise concerns about the adequacy of current artisanal post-harvest practices, such as open-air drying, which may be insufficient to ensure hygienic safety.

This underscores the urgent need to integrate optimized nutrient strategies with improved harvest timing and cost-effective sanitary interventions. Future research should focus on refining nutrient ratios, assessing alternative disinfection methods to reduce microbial contamination, evaluating community-level fermentation outcomes for enhanced product safety, and developing scalable, hygienic cultivation models suitable for rural production systems in sub-Saharan Africa.

## Figures and Tables

**Table 1:** Mineral composition of the initial inoculation medium and daily supplementation regimes per 1000 L culture basin.

Mineral (Chemical Compound)	Initial Inoculation (g/L)	Daily Supplementation (g/day)		
		Composition		
		A	B	C
Sodium bicarbonate (NaHCO_3_)	8	200	250	290
Sodium chloride (NaCl)	5	/	/	/
Potassium nitrate (KNO_3_)	2	40	/	30
Potassium sulfate (K_2_SO_4_)	1	/	10	/
Urea (CO(NH_2_) _2_)	/	35	40	/
Monopotassium phosphate (KH_2_PO_4_)	0.2	5	6	7
Magnesium sulfate (MgSO_4_·7H_2_O)	0.2	5	6	7
Calcium hydroxide (Ca (OH)_2_)	/	/	/	3
Calcium chloride (CaCl_2_·2H_2_O)	0.1	2	2	/
Ferrous sulfate (FeSO_4_·7H_2_O)	0.008	5	5	10

**Table 2:** Composition of culture media used for microbiological analyses.

Component	PCA (g/L)	SS Agar (g/L)	MacConkey Agar (g/L)
Beef extract/infusion	/	5.0	/
Peptone (casein and gelatin digest)	5.0	2.5 (Casein), 2.5 (Animal tissue)	1.5 (Casein), 17 (Gelatin), 1.5 (Tissue)
Yeast extract	2.5	/	/
Glucose	1.0	/	/
Lactose	/	10.0	10.0
Bile salts	/	8.5	1.5
Sodium citrate	/	8.5	/
Sodium thiosulfate	/	8.5	/
Ferric citrate	/	1.0	/
Sodium chloride (NaCl)	/	/	5.0
Neutral red	/	0.025	0.03
Crystal violet	/	/	0.001
Brilliant green	/	0.00033	/
Agar	15.0	13.5	13.5
Final pH (25 °C)	7.0 ± 0.2	7.2 ± 0.2	7.1 ± 0.2

Plate Count Agar (PCA), *Salmonella*–*Shigella* Agar (SS Agar).

**Table 3:** Physicochemical parameters.

Parameters	Mean ± SD	Min	Max
Temperature (°C)	23.45 ± 0.80	21.4	24.9
Density (g/L)	1015.87 ± 2.39	1012	1021
pH	10.00 ± 0.10	10.0	10.5
Secchi (cm)	4.57 ± 1.11	3.0	7.0
Salinity (g/L)	20.16 ± 2.03	17.0	24.1

Standard deviation (SD), Minimum (Min), Maximum (Max).

**Table 4:** Proximate composition for Spirulina grown under different nutrient regimes on Days 10, 20, and 30.

Experiment	Day	Moisture (%)	Proteins (%)	Carbohydrates (%)	Lipids (%)	Ash (%)
Control	10	6.85 ± 0.07	44.80 ± 0.65	20.30 ± 0.44	1.50 ± 0.10	19.10 ± 0.30
	20	6.70 ± 0.05	47.25 ± 0.58	22.15 ± 0.51	1.65 ± 0.12	19.30 ± 0.28
	30	6.60 ± 0.04	49.20 ± 0.55	24.10 ± 0.47	1.73 ± 0.09	19.55 ± 0.21
Composition A	10	**5.10 ± 0.06**	**51.80 ± 0.70**	**14.00 ± 0.35**	**3.20 ± 0.18**	18.90 ± 0.33
	20	**4.85 ± 0.04**	**56.20 ± 0.62**	**15.40 ± 0.28**	**3.10 ± 0.15**	19.40 ± 0.30
	30	**4.67 ± 0.00**	**60.38 ± 0.68**	**16.80 ± 0.23**	**3.07 ± 0.19**	19.75 ± 0.35
Composition B	10	6.65 ± 0.09	**47.00 ± 0.61**	**18.80 ± 0.40**	1.95 ± 0.13	18.95 ± 0.32
	20	6.45 ± 0.08	**51.00 ± 0.70**	21.10 ± 0.30	1.85 ± 0.10	19.30 ± 0.26
	30	6.25 ± 0.11	**54.87 ± 0.68**	23.67 ± 0.32	1.77 ± 0.05	19.69 ± 0.27
Composition C	10	6.90 ± 0.06	**42.50 ± 0.55**	**22.90 ± 0.52**	1.55 ± 0.18	19.50 ± 0.30
	20	6.80 ± 0.05	**46.20 ± 0.60**	**25.30 ± 0.45**	1.42 ± 0.14	19.75 ± 0.22
	30	6.67 ± 0.00	**50.51 ± 0.65**	**28.02 ± 0.41**	1.36 ± 0.23	20.11 ± 0.16

Data are mean ± SD; bold values indicate significant differences compared to the control at *p* < 0.05.

**Table 5:** Microbial contamination levels in Spirulina cultivated under different nutrient regimes over time.

			MacConkey Agar		SS Agar	
Experiment	Day	Total *Mesophilic* Counts (CFU/g)	*E. coli* (CFU/g)	*Salmonella*/*Shigella* (×10^2^ CFU/g)	*Salmonella* (×10^2^ CFU/g)	*Shigella* (×10^2^ CFU/g)
Control	10	3.2 ± 0.45 × 10^5^	0.42 ± 0.12 × 10^3^	1.03 ± 0.29	0.61 ± 0.14	0.42 ± 0.12
	20	4.1 ± 0.67 × 10^6^	0.65 ± 0.22 × 10^3^	1.53 ± 0.37	0.91 ± 0.24	0.62 ± 0.20
	30	4.7 ± 0.89 × 10^6^	0.81 ± 0.21 × 10^4^	1.92 ± 0.45	1.20 ± 0.35	0.72 ± 0.24
Composition A	10	3.4 ± 0.51 × 10^5^	0.47 ± 0.18 × 10^3^	1.12 ± 0.41	0.70 ± 0.27	0.42 ± 0.16
	20	4.2 ± 0.66 × 10^6^	0.63 ± 0.20 × 10^3^	1.63 ± 0.56	1.07 ± 0.30	0.56 ± 0.25
	30	5.0 ± 0.32 × 10^6^	0.805 ± 0.235 × 10^4^	2.07 ± 1.02	2.07 ± 0.71	0.30 ± 0.40
Composition B	10	3.8 ± 0.77 × 10^5^	0.72 ± 0.25 × 10^3^	2.20 ± 0.60	1.30 ± 0.44	0.90 ± 0.35
	20	4.4 ± 1.10 × 10^6^	0.92 ± 0.28 × 10^3^	3.22 ± 0.97	2.10 ± 0.53	1.12 ± 0.56
	30	4.5 ± 1.47 × 10^6^	1.05 ± 0.075 × 10^4^	4.04 ± 1.33	4.09 ± 1.81	5.92 ± 3.15
Composition C	10	4.1 ± 0.68 × 10^5^	0.61 ± 0.27 × 10^3^	1.53 ± 0.58	0.80 ± 0.33	0.73 ± 0.30
	20	5.0 ± 1.12 × 10^6^	0.85 ± 0.32 × 10^3^	1.83 ± 0.69	0.90 ± 0.39	0.93 ± 0.41
	30	6.1 ± 4.29 × 10^6^	1.07 ± 1.01 × 10^4^	2.00 ± 1.78	0.40 ± 0.35	1.05 ± 1.43

Data are mean ± SD; indicate significant differences compared to the control at *p* < 0.05.

## Data Availability

The original contributions presented in the study are included in the article, further inquiries can be directed to the corresponding authors.
